# Electrochemical control of [FeFe]-hydrogenase single crystals reveals complex redox populations at the catalytic site†

**DOI:** 10.1039/d1dt02219a

**Published:** 2021-07-13

**Authors:** Simone Morra, Jifu Duan, Martin Winkler, Philip A. Ash, Thomas Happe, Kylie A. Vincent

**Affiliations:** Department of Chemistry, University of Oxford, Inorganic Chemistry Laboratory South Parks Road Oxford OX1 3QR United Kingdom kylie.vincent@chem.ox.ac.uk; Faculty of Biology and Biotechnology, AG Photobiotechnology, Ruhr-University Bochum 44801 Bochum Germany

## Abstract

Elucidating the distribution of intermediates at the active site of redox metalloenzymes is vital to understanding their highly efficient catalysis. Here we demonstrate that it is possible to generate, and detect, the key catalytic redox states of an [FeFe]-hydrogenase in a protein crystal. Individual crystals of the prototypical [FeFe]-hydrogenase I from *Clostridium pasteurianum* (CpI) are maintained under electrochemical control, allowing for precise tuning of the redox potential, while the crystal is simultaneously probed *via* Fourier Transform Infrared (FTIR) microspectroscopy. The high signal/noise spectra reveal potential-dependent variation in the distribution of redox states at the active site (H-cluster) according to state-specific vibrational bands from the endogeneous CO and CN^−^ ligands. CpI crystals are shown to populate the same H-cluster states as those detected in solution, including the oxidised species Hox, the reduced species Hred/HredH^+^, the super-reduced HsredH^+^ and the hydride species Hhyd. The high sensitivity and precise redox control offered by this approach also facilitates the detection and characterisation of low abundance species that only accumulate within a narrow window of conditions, revealing new redox intermediates.

## Abstract 2

The distribution of redox species in single crystals of [FeFe]-hydrogenase can be tuned electrochemically. All catalytic intermediates are observed by simultaneous infrared microspectroscopy, providing a roadmap for enriching specific redox states.

## Graphical abstract

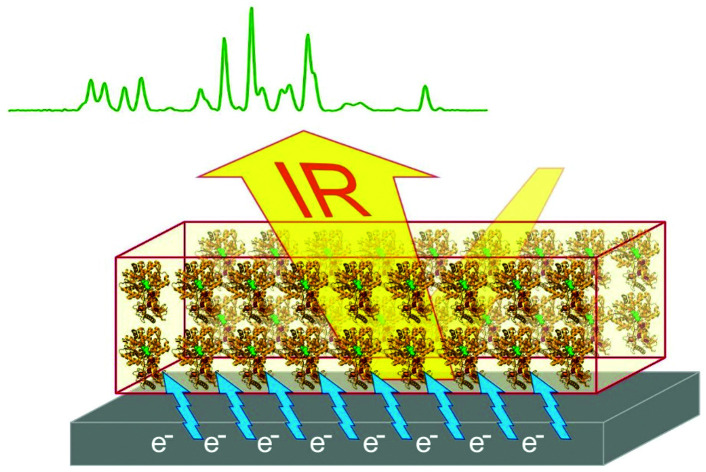

## Introduction

[FeFe]-hydrogenases are redox active metalloenzymes that reversibly catalyse the reduction of two protons (H^+^) to H_2_ at high turnover rates *via* the H-cluster, a highly specialised iron sulfur centre.^1–3^

**Fig. 1:**
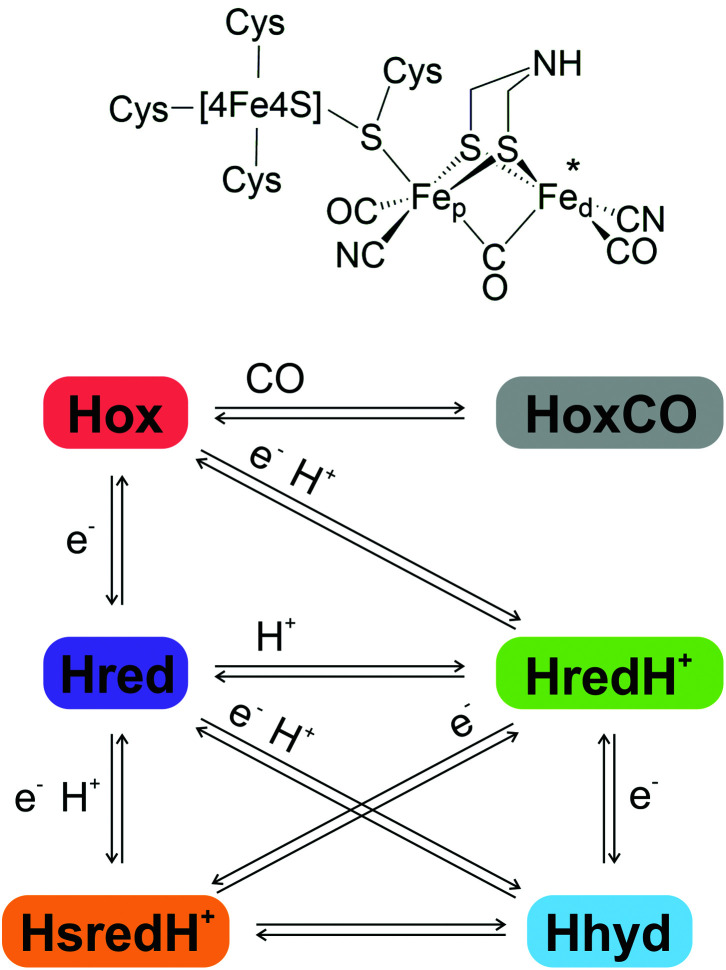
Top, schematic representation of the H-cluster structure in the oxidised Hox state. Bottom, schematic representation of the principal H-cluster redox states and the electron and proton transfer events leading to corresponding state transitions.

The H-cluster (Fig. 1, top) comprises a cubane [4Fe4S] sub-cluster, coordinated by four conserved cysteine residues, one of which bridges to a [2Fe] sub-cluster. The [2Fe]-cluster is coordinated by three CO, two CN^−^ ligands and an azadithiolate (adt) ligand in a bridging position between the Fe sites, which are differentiated as proximal and distal (Fe_p_ and Fe_d_) according to their relative proximity to the [4Fe4S] cluster.^4–6^

The unique catalytic features of [FeFe]-hydrogenases arise from the finely tuned synergy between the H-cluster and its evolutionarily optimised protein environment.^7–12^ Taking information from spectroscopy, structural studies and computational methods, several potential catalytic intermediates (Fig. 1, bottom) have been described and partially characterised. Their relevance for the catalytic cycle and their exact atomic structure are still under investigation.^13–15^

It is widely accepted that the H-cluster in the oxidised ‘ready’ state, Hox, displays a [4Fe4S]^2+^-Fe(ii)Fe(i) configuration, with one of its carbonyl ligands in a bridging position between the two metal atoms (μCO), and a vacant axial coordination site at the distal iron atom (marked by an asterisk in Fig. 1).^16^ Reversible binding of an additional CO molecule to Hox results in HoxCO, an inhibited state of the H-cluster.^17–19^

A 1-electron reduction of the H-cluster in Hox yields the reduced state Hred that features a similar geometry with a [4Fe4S]^1+^-Fe(ii)Fe(i) configuration.^13,20,21^ An alternative nomenclature refers to this species as *Hred*′.^15,22^

The protonated reduced state HredH^+^ (*Hred* according to the alternative nomenclature) displays a [4Fe4S]^2+^-Fe(i)Fe(i) configuration and can be obtained either by a single proton binding to Hred (with simultaneous intra-cluster electron transfer from [4Fe4S] to Fe_p_), or by proton-coupled electron transfer (PCET) from Hox. The exact location of the additional proton, and the reconfiguration of the bridging CO to a terminal position, are currently debated: there is disagreement as to whether the proton remains attached to the nitrogen atom of the azadithiolate bridge or shifts to a location between the two iron atoms forming a bridging hydride (μH^−^). Correspondingly it is likewise unclear whether the μCO ligand remains in the bridging position or shifts towards Fe_d_, adopting an axial configuration. Accordingly, the catalytic and mechanistic relevance of HredH^+^ is under intense debate.^13,14,20,23,24^

A second 1-electron reduction step may generate the super-reduced state HsredH^+^, exhibiting a [4Fe4S]^1+^-Fe(i)Fe(i) configuration.^25^ Also in this case, consensus is lacking on the protonation state, the actual geometry, and the relevance for catalytic turnover.^14^

Alternatively, the second 1-electron reduction step may generate the key catalytic intermediate Hhyd.^26–29^ Hhyd has been identified only recently, thanks to genetic alteration of specific conserved amino acids which was found to perturb the distribution of the H-cluster redox states.^7,26,28^ There is agreement that Hhyd exhibits a [4Fe4S]^1+^-Fe(ii)Fe(ii) configuration with an axial hydride ligand and a bridging CO. More recently, it has been proposed that the second proton required to complete H_2_ synthesis may transiently bind to the azadithiolate bridge, resulting in the intermediate HhydH^+^ with a configuration of [4Fe4S]^2+^-Fe(i)Fe(ii).^30,31^

Separate protonation events have been proposed to take place on the cubane sub-cluster of the catalytic centre, resulting in a duplicate set of alternatively protonated redox states which have been termed HoxH, HoxHCO, Hred′H and HhydH.^15,21,22,32^

The H-cluster redox states and reactivity have been extensively investigated in model enzymes, particularly CrHydA1 (from the green alga *Chlamydomonas reinhardtii*), CpI (from the anaerobic nitrogen-fixing bacterium *Clostridium pasteurianum*) and DdH (from the sulfate-reducing bacterium *Desulfovibrio desulfuricans*).^2,3,33^ CaI (from the solvent-producer *Clostridium acetobutylicum*) has a high degree of homology with CpI and has likewise been characterised.^14,34^ The main difference between these enzymes is their domain composition: CrHydA1 consists of a single domain hosting the H-cluster only, while DdH and CpI/CaI include additional iron sulfur centres (2 and 4, respectively) which act as electron relay chains (commonly referred to as the F-domain).

Generating [FeFe]-hydrogenase crystals enriched in specific redox states may allow significant progress towards elucidating the exact structure of the key reaction intermediates by X-ray diffraction,^35^ but obtaining a sufficiently high level of homogeneity for individual redox states is challenging.^36^

The use of chemical oxidants or reductants to modulate the redox state in crystals is convenient for simple proteins but has limitations when a complex catalytic cycle with many possible intermediates is involved, as with [FeFe]-hydrogenases. These intermediates usually coexist in a dynamic equilibrium at proportions defined by the given system parameters.

Indeed, there have been only two reports of reduced [FeFe]-hydrogenase crystal structures. In the first case, DdH was treated with high pressure H_2_ and reduction of the crystal was demonstrated by UV-VIS spectroscopy, but this did not provide exact information regarding the precise redox configuration of the H-cluster.^37^ In the second case, CpI was treated with sodium dithionite as reducing agent, but no spectroscopic evidence was presented to verify or characterise the H-cluster state.^38^

**Fig. 2:**
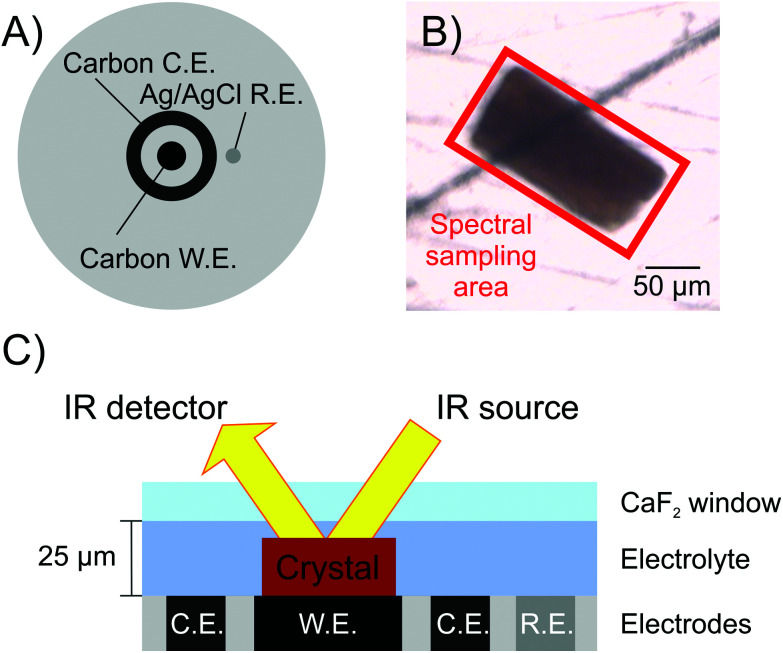
The spectroelectrochemical cell used in this study features a 3-electrode system with mirror-polished glassy carbon working electrode that simultaneously allows for electrochemical control of the protein crystal and detection by IR spectroscopy in reflection mode. (A) top view of the cell. (B) a representative CpI crystal as seen by visible microscopy, highlighting the area sampled by IR spectroscopy. (C) Schematic cross-section view of the cell (not to scale), including working electrode (W.E.), counter electrode (C.E.) and reference electrode (R.E.). The electrodes are embedded in a Delrin base plate.

In order to investigate what redox states of the H-cluster can be populated in [FeFe]-hydrogenase crystals, and how this process can be precisely controlled, we extend the previous work carried out on single crystals of a [NiFe]-hydrogenase.^39^ Here we show that it is possible to apply electrochemical control to single crystals of [FeFe]-hydrogenase CpI, with simultaneous *in crystallo* monitoring of the H-cluster speciation by infrared (IR) microspectroscopy (Fig. 2). IR sampling is particularly well suited for this purpose because of its ability to specifically detect vibrational features of the intrinsic CO/CN^−^ ligands at the active site of hydrogenases, which are sensitive to changes in electron density at the metallo-site.

These results, in conjunction with our recent paper on the [NiFe]-hydrogenase, *E. coli* Hyd1,^40^ demonstrate that this technique has broader applicability to complex metalloenzymes.

## Experimental

### Preparation of CpI crystals

*Clostridium pasteurianum* hydrogenase I (CpI) was overexpressed in *E. coli*, artificially maturated *in vitro*, and crystallised according to previously described protocols.^32,41^ Crystals from the same batch as those used in this study yielded high resolution X-ray diffraction data, reaching resolution <2 Å. Diffraction data confirm the packing pattern previously reported for other structures of CpI with space group (*P*12_1_1) and almost identical unit cell dimensions.^41^

### IR microspectroscopy under electrochemical control

CpI crystals were gently resuspended in their mother liquor (22% PEG4000, 0.1 M MES buffer pH 6, 0.4 M MgCl_2_ and 18% glycerol) under the inert N_2_ atmosphere of an anaerobic glove box (Glove Box Technology, UK). For experiments performed at pH 8, crystals were resuspended in 0.1 M Tris buffer pH 8.0, likewise supplemented with 22% PEG4000, 0.4 M MgCl_2_ and 18% glycerol. For control experiments with CpI in solution, the buffer composition was either 0.1 M MES-NaOH (pH 6.0) or Tris-HCl (pH 8.0) supplemented with 0.1 M NaCl and the enzyme concentration was 0.7–0.9 mM.

All samples were further supplemented with a mixture of redox mediators to facilitate electron transfer between the working electrode and the protein crystal, covering the potential range between −600 and −200 mV *vs.* the Standard Hydrogen Electrode (SHE).^13,42^ The mixture consisted of 0.5 mM each Eu(iii) BAPTA (*E*_m_ = −650 mV), methyl viologen (*E*_m_ = −449 mV), benzyl viologen (*E*_m_ = −329 mV) and anthraquinone-2-sulfonate (*E*_m6_ = −180 mV; *E*_m8_ = −276 mV, where *E*_m6_ refers to the midpoint potential at pH 6.0 for example). Also, 0.4 mM reduced myoglobin (from equine skeletal muscle, Sigma) was added to scavenge any traces of O_2_.

Subsequently, the sample was transferred to a custom-built gas-tight spectroelectrochemical cell (Fig. 2) that was optimised from a previous report.^39^ In this cell, the sample sits on a flat surface featuring a mirror polished glassy carbon disk working electrode, a carbon ring counter electrode, and a leak-free Ag/AgCl reference electrode (Innovative Instruments Inc, USA). The cell is sealed by a CaF_2_ window (1.5 mm thickness, Crystran, UK) with a 25 μm PTFE spacer (Pike Technologies, USA). The reflection mode adopted requires the IR beam to pass through the crystal twice, doubling the sample path length. To simplify cross-reference to previous literature, potentials measured *vs.* the Ag/AgCl reference electrode were converted to mV *vs.* SHE by adding +209 mV, and all potentials mentioned in the text are quoted *vs.* SHE.

Electrochemical control was achieved *via* a Metrohm Autolab PGSTAT128N potentiostat controlled by NOVA software.

The equilibration time was tested in preliminary kinetics experiments (Fig. S1†) and fixed to 15 minutes at each potential in the full potential titrations.

FTIR spectra were recorded with a Bruker Vertex 70 equipped with a Hyperion 2000 microscope, a Globar source, 15× objective and a mercury cadmium telluride (MCT) detector. Spectra were recorded with a resolution of 2 cm^−1^ and averaged from 512 accumulations.

### Data analysis

Baseline corrected spectra were obtained by interpolating and subtracting in OriginLab 2017 a spline baseline to compensate for the contribution of water to the spectral region of interest (Fig. S2†). Difference spectra were obtained by directly subtracting raw spectra without further manipulation.

## Results and discussion

### CpI crystals are stable and the redox control is effective and fully reversible

**Fig. 3:**
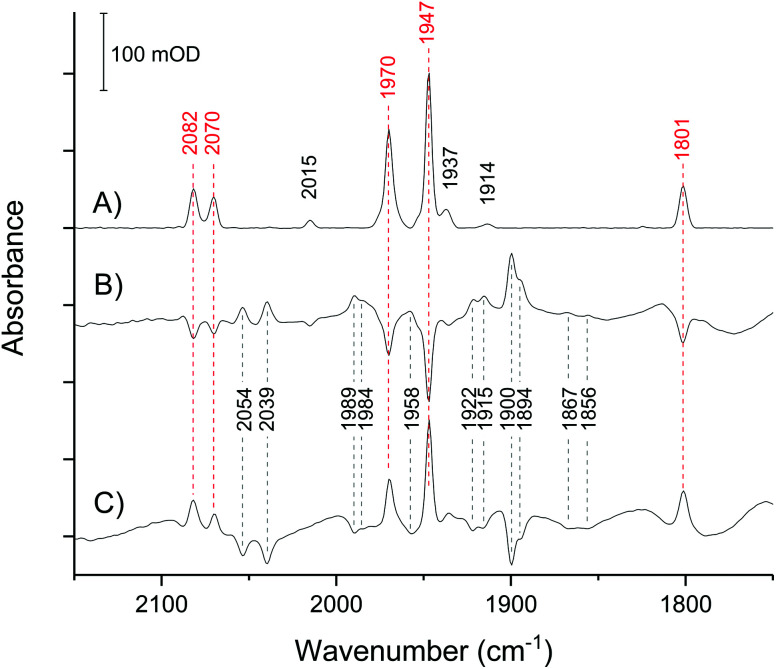
IR microspectroscopy of a single CpI crystal in its mother liquor. (A) spectrum of the untreated crystal with no applied potential (open circuit potential: approx. −200 mV). (B) Difference spectrum of the crystal after applying a potential of −367 mV (reduced *minus* oxidised). (C) Difference spectrum upon stepping back to −200 mV (re-oxidised *minus* reduced). Peaks marked by red wavenumber labels belong to the IR-signature of Hox.

In the absence of external electrochemical control, the H-cluster in CpI crystals equilibrates in a mostly homogeneous state (Fig. 3A), displaying intense IR peaks at 2082, 2070, 1970, 1947, 1801 cm^−1^, which are unambiguously assigned to Hox.^28,38,43^ Only a minor proportion of other states can be detected, as indicated by low intensity signals at 2015 (HoxCO), 1975 and 1953 (HoxH), 1937 (Hred) and 1914 cm^−1^ (HredH^+^). Traces of HoxCO are commonly observed in spectroscopic studies of [FeFe]-hydrogenases.^44^ Since the contribution of HoxCO is minimal in the crystal spectra, it will not be taken into account in the subsequent discussion.

The spectra have excellent signal-to-noise ratio and peak intensity up to 200 mOD, which is consistent with the high effective enzyme concentration within the crystal of approximately 9.4 mM as calculated on the basis of the space group and unit cell parameters.^41^ Moreover, the large size of CpI crystals makes it possible to obtain high quality spectra using a common Globar IR source, while working with smaller crystals of the [NiFe]-hydrogenase Hyd-1 from *Escherichia coli* required the use of synchrotron IR radiation, as seen in previous work.^39,40^

Microspectroscopy on various CpI crystals from the same batch shows variations in signal intensity consistent with differences in shape and size, but overall consistency in the distribution of redox states (Fig. S3†).

The crystal responds very quickly to an applied potential, with the equilibration time following a potential step being in the range of 2–5 minutes (Fig. S1†). Stepping from −200 mV to −367 mV causes loss of Hox and the increase of several peaks that reveal the accumulation of reduced species, with HredH^+^ (1900 cm^−1^) being the most abundant (Fig. 3B).

Importantly, all changes are fully reversible: stepping back to −200 mV results in direct reversal of the spectral changes (Fig. 3C). This clearly demonstrates that CpI in the crystal is stable in response to the applied potential.

### Validating the significance of the H-cluster chemistry in crystals

**Fig. 4:**
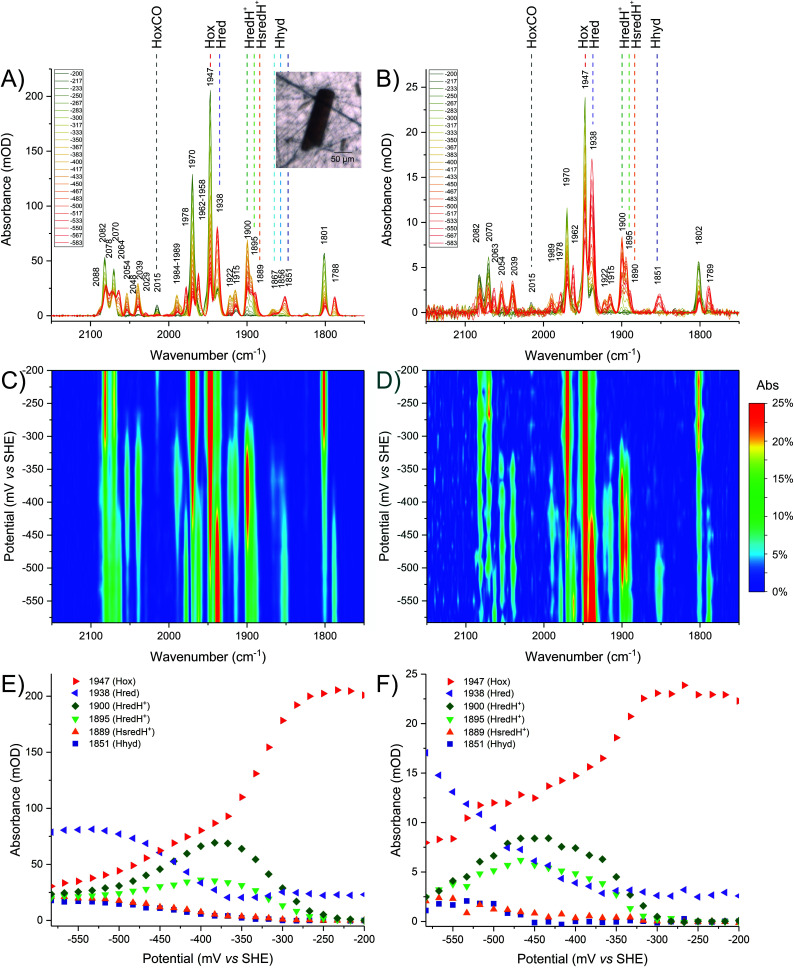
Comparison of redox titrations of CpI at pH 6, *in crystallo* (left column) and in solution (right column). The oxidised sample was reduced in steps from −200 mV to −583 mV. The sample was equilibrated for 15 minutes at each given potential. Each given potential is reported in the legend in mV. The top panels present baseline-corrected spectra obtained from (A) a CpI crystal or (B) a CpI solution. The inset shows a visible image of the crystal used. The middle panels present 2D plots of the titration for (C) the crystal or (D) the enzyme in solution. The bottom panels present titration plots for selected wavenumber positions representing each redox state for (E) the crystal or (F) the enzyme solution. For a detailed description of the peak assignments see the main text and refer to Table 1.

In order to validate the results observed in crystals, detailed redox (potential step) titrations were performed comparing CpI crystals (Fig. 4A, C and E) with CpI samples in solution (Fig. 4B, D and F). A high level of comparability was achieved by using the same IR microspectroscopy cell containing protein solution instead of crystals.

It should be noted that *wild type* CpI redox titrations have not been reported previously, but the data obtained in this work are consistent with conventional spectroelectrochemical studies of other [FeFe]-hydrogenases in solution, such as CrHydA1 and DdH.^11,13,45,46^

Lowering the potential from −200 to −583 mV in small steps (17 mV each) confirms that precise electrochemical control can be achieved, as demonstrated by a gradual decline of Hox signals while a variety of reduced species appear, including Hred, HredH^+^, Hhyd and HsredH^+^ (Fig. 4).

Also in this case, re-oxidation to −200 mV shows full reversibility of the process both in solution and *in crystallo*. No macroscopic damage to the crystal can be detected in visible images after the titration (Fig. S4†). Appearance of HoxCO can be an indicator of active site degradation;^44^ here, enzyme stability within individual crystals is further demonstrated by the fact that only a very low fraction of HoxCO is detectable while sweeping the potential back and forth during experiments over 12 hours.

In solution it is impossible to achieve the same H-cluster density as in the crystal; the protein has been concentrated to approximately 0.9 mM and, consequently, the signal intensity and signal-to-noise ratio are much lower. Despite this technical limitation, the redox titration of CpI in solution indicates very similar speciation as observed for the crystal (compare Fig. 4B with A).

The same redox species can be observed, as peak positions and relative peak intensity patterns are identical. The titration plots show remarkable similarity (compare Fig. 4D with C and Fig. 4F with E), with minor differences only detectable at potentials below the H^+^/H_2_ redox couple, where the relative distribution of reduced states is slightly different. This may be due to differences in accumulation of H_2_ and in the local concentration of protons. Both factors are expected to be particularly influential in the confined space between molecules in a densely packed protein crystal.

Moreover, when the same experiment is performed at pH 8, the data gained from crystallised and dissolved CpI show striking resemblance (Fig. S5†).

These results demonstrate that the functionality of CpI in the crystalline state is fully retained and supports the significance of the mechanistic implications described below.

### Dissecting the reduced states in CpI

Since previous IR peak assignments for CpI have only been made by comparison to already assigned spectra of other [FeFe]-hydrogenases,^28,32^ the identification and interpretation of some of the reduced species observed during redox titration experiments require a more detailed analysis, now being possible given the excellent signal-to-noise ratio of the *in crystallo* spectra.

As discussed below, on the basis of the redox titrations at different pH (namely 6 and 8, Fig. 4 and S5† respectively), several low intensity signals can be conveniently dissected here, resulting in an updated assignment of IR signatures for each redox state in CpI, as summarised in Table 1. For clarity in the figures and the discussion, the most isolated (*i.e.* non-overlapping) and intense peak was identified for each species and used as a unique marker (shown in bold in Table 1).

**Table 1:** Summary of the IR signatures for the H-cluster redox states in CpI. The peaks highlighted in bold are considered unique markers for each redox state and are used as a reference in the text and in titration plots (Fig. 4E and 4F)

Redox state	Peak position (cm^−1^)	
	CN ligands	CO ligands
Hox	2082, 2070	1970, **1947**, 1801
HoxH	NA, NA	1975, 1953, NA
HredH^+^ family	2054, 2039	1915, **1895**, NA
	2054, 2039	1922, **1900**, NA (additional H^+^)
Hhyd family	NA, NA	1978, 1962, **1851** (Hhyd:red)
	NA, NA	1984, NA, **1856** (HhydH^+^)
	NA, NA	1989, 1958, **1867** (Hhyd:ox)
Hred	2078, 2064	**1938**, 1788, NA
HsredH^+^	2048, 2029	**1889**, NA, NA
HoxCO	2090, 2076	**2015**, 1973, 1969, 1807

At pH 6, it is clear that the redox landscape can be divided into three regions (Fig. 4C and E): (1) at high potentials (*i.e.* −200 to −250 mV) the sample is essentially composed of Hox; (2) at moderately reducing potentials (*i.e.* −250 to −425 mV), Hox is converted into a complex mixture of multiple redox states that accumulates, reaches a maximum at −367 mV and then is depleted; (3) at more negative potentials (*i.e.* below approximately −425 mV) a new complex mixture of further reduced species is accumulated, replacing the previous pool. Spectra representative of each of these regions are presented in Fig. S7.†

The same features are observed in redox titrations at pH 8 (Fig. S5A, S5C and S5E†). All species appear with the same spectral signature and all redox transitions are shifted, as a result of the lower availability of protons and corresponding shifts in the proton-coupled redox equilibria. This demonstrates that CpI crystals efficiently equilibrate when being soaked in buffers with a modified composition.

### The HredH^+^ family

Within the moderate reduction potential range (see also Fig. S8A†), four main peaks reach a maximum abundance at −367 mV and are readily assigned to HredH^+^ (2054, 2039, 1915 and 1900 cm^−1^). It is impossible to unambiguously locate the fifth peak, as this is expected to be overlapping with more intense signals from Hox (either in the region around 1970 or 1800 cm^−1^) and thus it is difficult to further comment on the coordination state of μCO in the HredH^+^ state solely on the basis of the presented IR data. Future work at cryogenic temperature may help to clarify this aspect by analogy to recent research performed on [FeFe]-hydrogenases in frozen solution.^14,24,47^

**Fig. 5:**
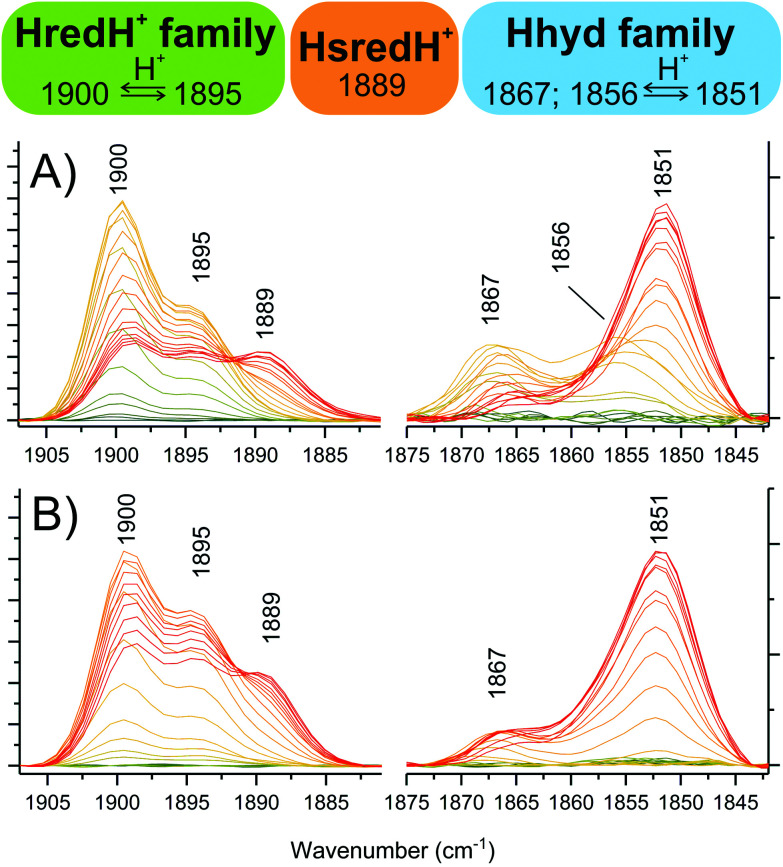
Dissecting the protonated reduced states in CpI crystals. Zooming into specific spectral regions reveals marker peaks associated with single species. (A) from titrations at pH 6. (B) from titrations at pH 8. Colour legend is the same as Fig. 4A.

Interestingly, peaks at 2039, 1922 and 1895 cm^−1^ (that were previously suggested to correspond to HsredH^+^ in CpI)^28^ coexist with HredH^+^ (1900 cm^−1^) signals, precisely following the same potential dependence plot (Fig. 4E and S6C†) and being depleted at more negative potentials (*i.e.* lower than −500 mV). Because of the similar redox behaviour and similar wavenumber position (marker peaks 1900 and 1895 cm^−1^), we propose that these signals belong to another version of HredH^+^ and that these two related species could be classified as a “family” (Fig. 5).

The two related redox species might only differ in minor ways, such as protonation state. For example, Ratzloff *et al.*^14^ proposed that a 1894 cm^−1^ species in the structurally related [FeFe]-hydrogenase CaI represents an unprotonated form of HredH^+^ (1899 cm^−1^ in CaI) with the same formal electron configuration [4Fe4S]^2+^-Fe(i)Fe(i). Alternatively, computational studies by Senger *et al.*^22^ suggested that further protonation events at a cysteine coordinating the cubane sub-cluster may result in IR signatures of the H-cluster being collectively blue-shifted by 4–8 cm^−1^.

It should also be noted that in earlier studies of CrHydA1 and DdH a single HredH^+^ species was consistently reported,^11,13^ but recent time-resolved studies of CrHydA1 revealed a similar level of complexity in this spectral region with two species evident with peak maxima at 1891 and 1896 cm^−1^.^48^

Further evidence for a differential protonation state of these two related species is provided by comparing the relative abundance of each during titrations at different pH values (pH 6 *vs.* 8, Fig. 5). In both cases, marker peaks 1900 and 1895 cm^−1^ (and the other peaks associated with the signature) coexist and follow the same titration plot, with maximum abundance at −367 mV at pH 6 or −450 mV at pH 8 (compare Fig. 4E and Fig. S5E†). However, the relative proportion of the two species is different: at low pH, the signal at 1900 cm^−1^ is significantly more intense relative to the other one (*A*_1900_/*A*_1895_ is 2.03 at pH 6 and 1.38 at pH 8), and a similar difference is observed when titrating CpI in solution (Fig. 4B and S5B†). This suggests that the species represented by the peak at 1900 cm^−1^ is more protonated than the one with the 1895 cm^−1^ marker band (Fig. 5). This interpretation is also in agreement with previous reports on CaI.^14^

### The Hhyd family

The other low intensity sets of signals observed in the moderate reduction potential range (maximum abundance at −367 mV) of the titration at pH 6 (*i.e.* 1989–1958–1867 and 1984–1856 cm^−1^) are in good agreement with previous reports on Hhyd, and they are all depleted at more negative potentials (below −400 mV), being converted into the more intense signature 1978–1962–1851 cm^−1^. Peaks arising from the cyanide ligands cannot be unambiguously assigned due to overlap between Hox and Hred signals in the region between 2065–2085 cm^−1^.

We propose to classify these three IR signatures as a “family” of Hhyd species (Fig. 5). Small spectral differences here might arise from minor structural variations, such as a different protonation state/position or from deviations in the electronic configuration such as a reduced or oxidised [4Fe4S] cluster.

The existence of multiple Hhyd species has also been proposed before: three CO stretching IR bands were originally detected for the C169S variant of CrHydA1 (1874, 1868, 1860 cm^−1^).^7^ More recently, a similar degree of diversity has been reported in *wild type* CrHydA1: two species were detected *in vivo* (1875, 1860 cm^−1^)^31^ and three species were differentiated at cryogenic temperatures (1865, 1861, 1851 cm^−1^).^49^ Furthermore, two different Hhyd species have been observed in *wild type* CaI (1856, 1852 cm^−1^);^14^ and CpI (1870, 1856 cm^−1^).^32^

On this basis we assign the most abundant species (associated with the 1851 cm^−1^ peak) to “conventional” Hhyd^26–29^ (also known as Hhyd:red)^49^ and the 1867 cm^−1^ species to Hhyd:ox (configuration [4Fe4S]^2+^-Fe(ii)Fe(ii)).^49^ Interestingly, Hhyd:ox is highly similar to a new species which was denoted HredH^+^ in a recent computational study.^50^ In CpI crystals, the 1856 cm^−1^ species is completely undetectable at pH 8 (Fig. 5 and Fig. S5†), suggesting an assignment for this species as the more protonated state HhydH^+^. Consistently, previous experiments with CpI in solution at pH 4 only displayed this spectral signature.^28,32^

Despite significant accumulation of numerous reduced species possibly differing in protonation state, we do not observe significant accumulation of HoxH and this is consistent with previous reports that this species accumulates only at low pH (≤6) and exclusively in the presence of sodium dithionite.^22^

A notable observation is that *wild type* CpI (both *in crystallo* and in solution) readily accumulates a relevant amount of Hhyd species under electrochemical control and mild pH conditions. To the best of our knowledge, this is the first time that Hhyd has been observed under such experimental conditions. Hhyd was undetectable in previous spectroelectrochemical solution-based experiments with CrHydA1^13,46^ or DdH,^11,45^ and could only be detected at very low pH^28^ or in enzyme variants with impaired proton reduction activity.^26,30,32^ The only other case of a *wild type* [FeFe]-hydrogenase accumulating Hhyd at pH 8 is CaI when treated with dithionite.^14^ The different propensity to accumulate Hhyd species will be investigated in future by systematically comparing different [FeFe]-hydrogenases and variants under the same experimental conditions.

### Hred and HsredH^+^ assignment

In addition to Hhyd species, at more negative potentials several other peaks arise (see also Fig. S8B†). Considering the titration plots and on a comparative basis, these can be assigned to Hred (2078, 2064, 1938 and 1788 cm^−1^) and HsredH^+^ (2048, 2029 and 1889 cm^−1^). Here again, it is impossible to unambiguously assign all five peaks for each redox state, as the missing peaks overlap with other more intense peaks in the crowded regions of the spectrum (*i.e.* 1940–1990 and/or 1780–1800 cm^−1^).

The accumulation of HsredH^+^ follows a similar trend at pH 6 and pH 8, where it appears at approximately −433 mV and then increases at lower potentials (Fig. 4E, S5E† and Fig. 5). This provides unambiguous assignment of the 1889 cm^−1^ marker peak to HsredH^+^, improving the previous characterisation of this species in clostridial hydrogenases.^14,28,32^

The titration plots of Hred vary significantly at different pH values. At pH 6, HredH^+^ reaches a maximum at −367 mV and then decreases, while Hred is most abundant below −533 mV (Fig. 4); at pH 8, HredH^+^ abundance reaches a maximum at −450 mV, while Hred is most abundant at −417 mV (Fig. S5†). Also in this case, there is significant agreement between experiments performed *in crystallo* and in solution. The counterintuitive formation of Hred at potentials lower or higher than HredH^+^, as a function of pH, is in marked contrast with previous studies on CrHydA1,^13^ where they coexist at the same potentials, but it correlates extremely well with studies on DdH.^11,21^ The fact that CpI features this behaviour is likely due to redox anti-cooperativity between the H-cluster and the proximal F-cluster (present in both CpI and DdH but absent in CrHydA1).

Some of the IR signals discussed above have been proposed to be due to the incorporation of a degraded form of the synthetic precursor [2Fe]^adt^ in DdH (namely 1988 and 1921 cm^−1^).^11^ However, since these peaks are redox active in our titrations and, more importantly, they were previously observed in CaI produced *via in vivo* maturation with the maturases HydEFG,^14,34^ it is more likely that they have a functional role.

## Conclusions

In summary, this work demonstrates that it is possible to control, electrochemically, the redox state of [FeFe]-hydrogenase crystals, and to probe the H-cluster simultaneously by IR microspectroscopy. Numerous reduced states can be generated *in crystallo*, showing that it is possible to cycle through all catalytically relevant states: Hox, Hred, HredH^+^, Hhyd and HsredH^+^.

Control experiments performed on the enzyme in solution display the same spectral features, demonstrating that CpI behaves in the same way when it is tightly packed in the ordered crystal structure, further supporting the significance of previous and future structural studies. Furthermore, the accumulation of complex redox mixtures upon reduction highlights the importance of probing the redox composition of [FeFe]-hydrogenase crystals when performing structural diffraction studies.

The high sensitivity of this technique, and the possibility to finely tune the redox state by changing the potential in a highly controlled manner allow for the detection and discrimination of species that only exist within a narrow window of pH and potential conditions. This has allowed the identification and assignment of Hhyd:red (1851 cm^−1^), HhydH^+^ (1856 cm^−1^), Hhyd:ox (1867 cm^−1^) in CpI, in addition to a new alternative version of HredH^+^ (1895 cm^−1^) that is characterised here for the first time. Moreover, an unambiguous assignment for HsredH^+^ in CpI was obtained (1889 cm^−1^).

In accordance with recent reports by others, our data show that various alternative redox states with similar spectral signatures coexist in [FeFe]-hydrogenases (families of states), these could be due to changes in the protonation state and location, the long-range effects of the F-cluster redox state, or other fine local structural changes.

This work provides a roadmap for enriching and confirming the presence of specific redox states in [FeFe]-hydrogenase crystals.

## Author contributions

S.M. performed the spectroelectrochemical experiments and analysed the data. J.D., M.W. and T.H. provided CpI samples and crystals. S.M., P.A.A. and K.A.V. conceived the study, acquired funding and wrote the manuscript. K.A.V. and T.H. provided supervision and access to equipment. All authors participated in manuscript preparation and revision. The final manuscript has been read and approved by all authors.

## Conflicts of interest

There are no conflicts to declare.

## Supplementary Material

DT-050-D1DT02219A-s001
