# Supplementary material for: Electrochemical control of [FeFe]-hydrogenase single crystals reveals complex redox populations at the catalytic site
Source: Dalton Trans. 2021 Jul 13;50(36):12655–63. doi: 10.1039/d1dt02219a (PMC8453692; doi:10.1039/d1dt02219a)
Supplement: DT-050-D1DT02219A-s001 [file DT-050-D1DT02219A-s001.pdf]

Electronic Supplementary Material (ESI) for Dalton Transactions.
This journal is © The Royal Society of Chemistry 2021

Electrochemical control of [FeFe]-hydrogenase single crystals reveals complex redox populations at the catalytic site

Simone Morra, Jifu Duan, Martin Winkler, Philip A. Ash, Thomas Happe, and Kylie A. Vincent

Electronic Supplementary Information (ESI)

Table of contents

Figure S1. Evaluation of equilibration time after a potential step.

Figure S2. Representative example of baseline correction procedure for IR spectra of CpI crystals.

Figure S3. FTIR microspectroscopy carried out on different crystals of CpI from the same batch.

Figure S4. Reversibility and sample stability.

Figure S5. Comparison of redox titrations of CpI at pH 8, *in crystallo* and in solution.

Figure S6. Titration plots for every wavenumber position as assigned to their respective species.

Figure S7. Spectra representative of the three regions that can be identified in the titration of CpI crystals at pH 6.

Figure S8. Difference spectra from raw data.

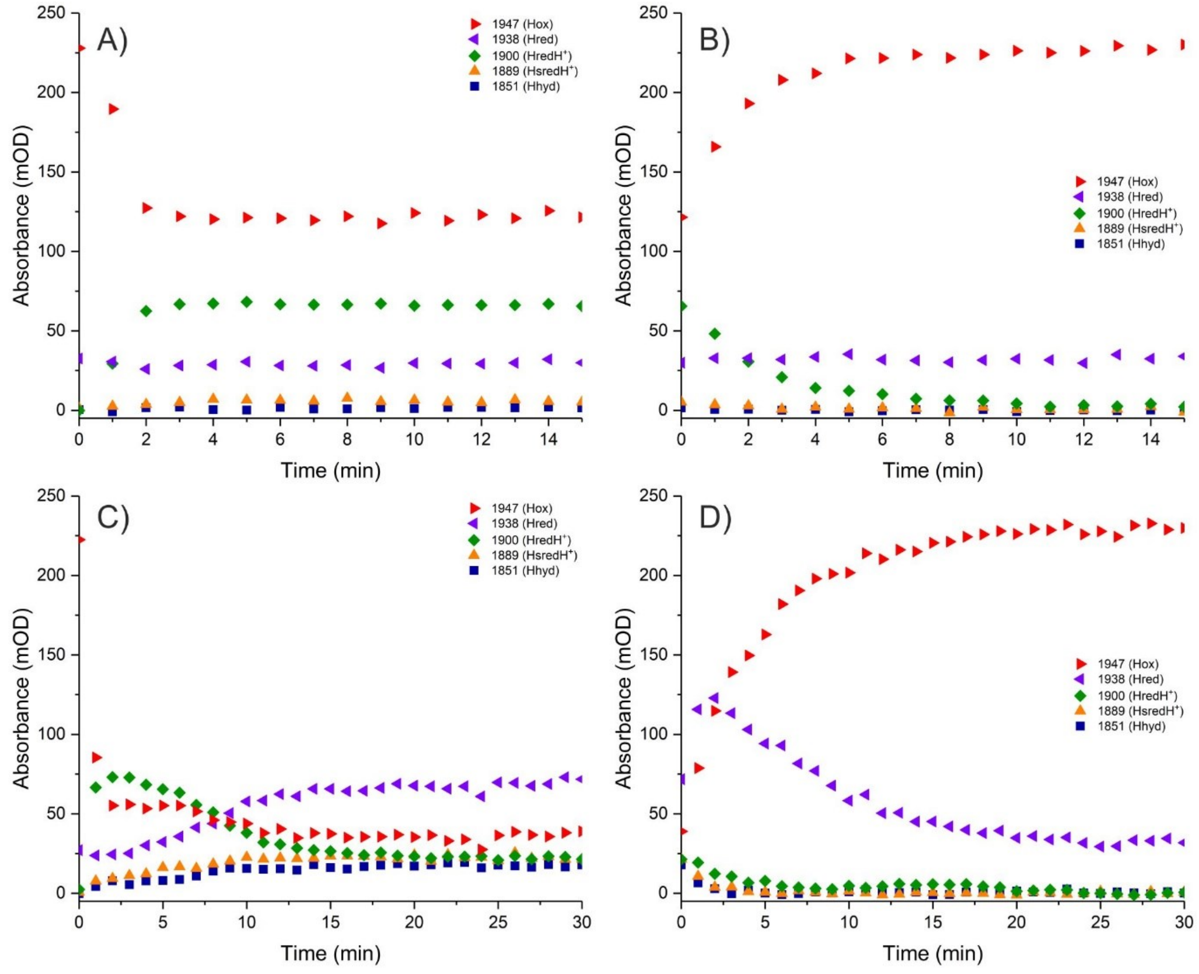

Figure S1. Evaluation of equilibration time after a potential step (crystal pH 6). Following the application of a potential step, spectra were acquired every 60 seconds (75 accumulations only per spectrum), and the abundance of each species was plotted against time. A) Following a step from -200 to -367 mV. B) Following a step from -367 mV back to -200 mV. C) Following a step from -200 mV to -667 mV. D) Following a step from -667 mV back to -200 mV. The equilibration time lies in the time frame of minutes. The equilibration time is shorter for smaller potential steps (*e.g.* -200/-367 mV) than for larger steps (*e.g.* -200/-667 mV), reflecting the larger number of consecutive transitions that are required to equilibrate over such a large potential range. Also, reduction takes place faster than re-oxidation, possibly due to local accumulation of H_2 that counterbalances the externally applied potential.

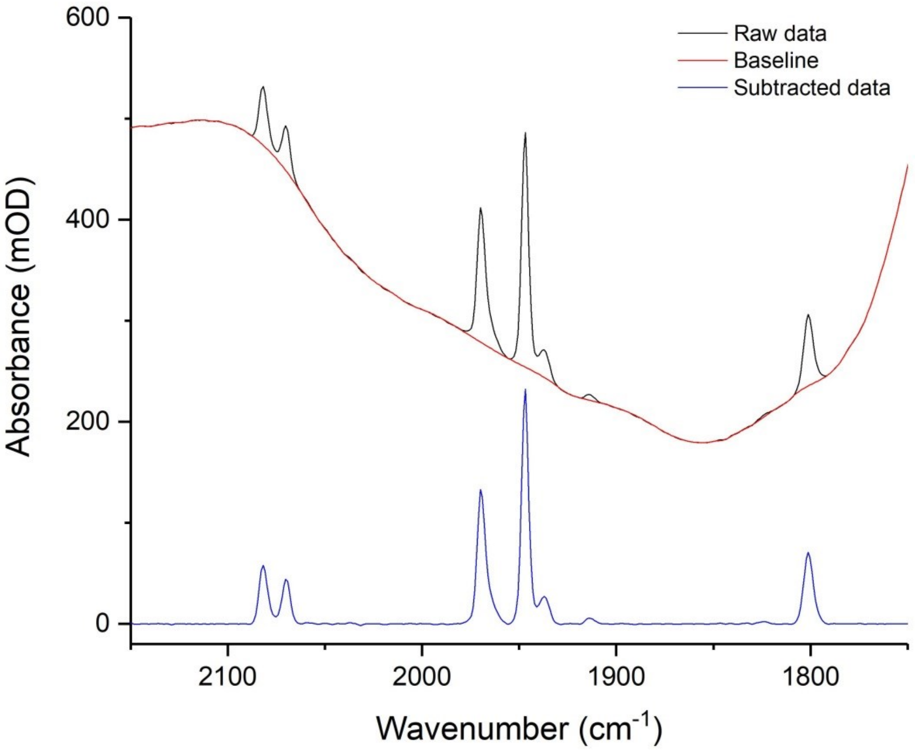

Figure S2. Representative example of baseline correction procedure for IR spectra of CpI crystals. The baseline was constructed in OriginPro 2017, by selecting anchor points in the regions of the spectrum where no peaks are present, as judged by second derivative analysis.

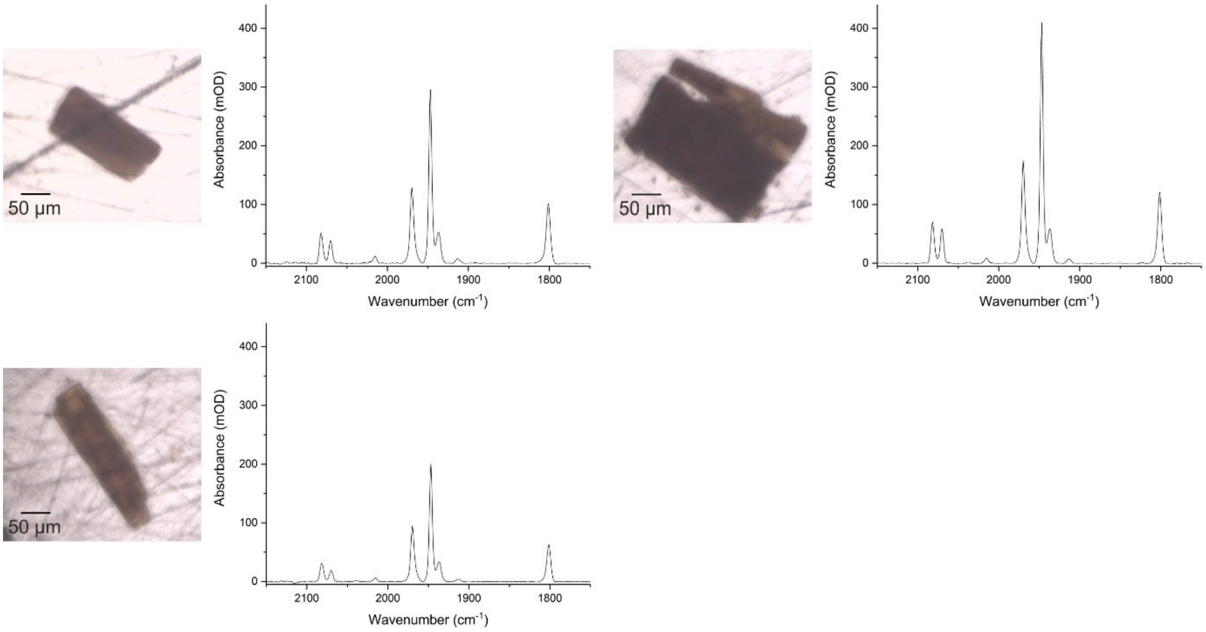

Figure S3. FTIR microspectroscopy carried out on different crystals of CpI from the same batch. Visible images of the crystals are shown to the left of each spectrum. Spectra were recorded without applied potential.

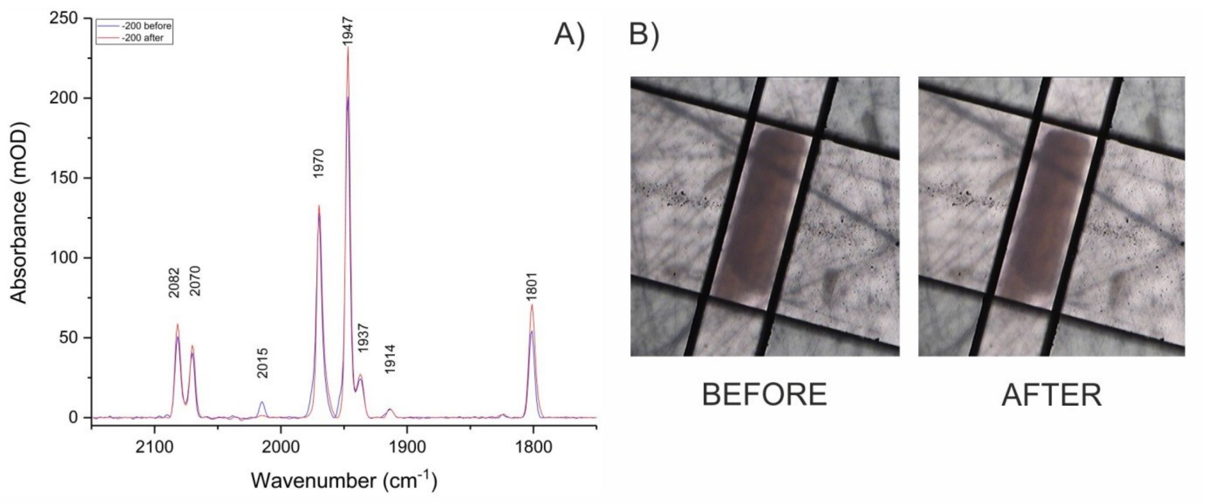

Figure S4. Reversibility and sample stability (pH 6). Despite the long duration of the full redox titration experiment (9-10 hours), CpI crystals do not show any visible sign of damage, and all IR spectral changes observed were reversible. A) The crystal was re-oxidised to -200 mV after the full titration. The minor proportion of HoxCO (2015 cm^{-1}) and $HoxH^{+}$ (1975 and 1953 cm^{-1}) that were found in the sample before the titration were converted back to Hox. B) Visible images of the crystal before and after the titration do not show any visible change.

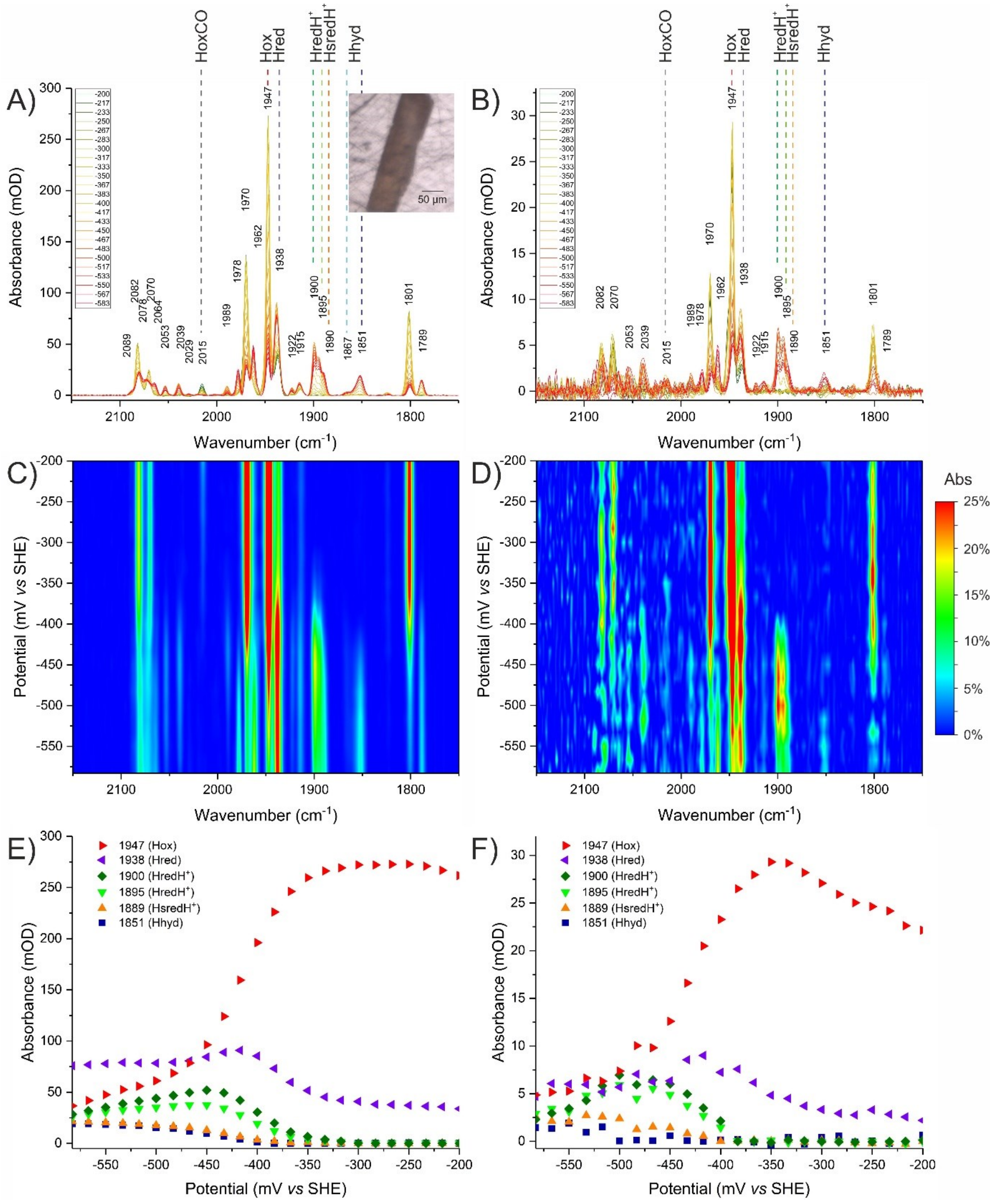

Figure S5. Comparison of redox titrations of CpI at pH 8, *in crystallo* (left column) and in solution (right column). Each given potential is reported in the legend as mV *vs* SHE. The top panels present baseline-corrected spectra obtained from A) a CpI crystal or B) a CpI solution. The inset shows a visible image of the crystal used. The middle panels present 2D plots of the titration for C) the crystal or D) the enzyme in solution. The bottom panels present titration plots for selected wavenumber positions representing each redox state for E) the crystal or F) the enzyme solution. For a detailed description of the peak assignment see the main text and refer to Table 1.

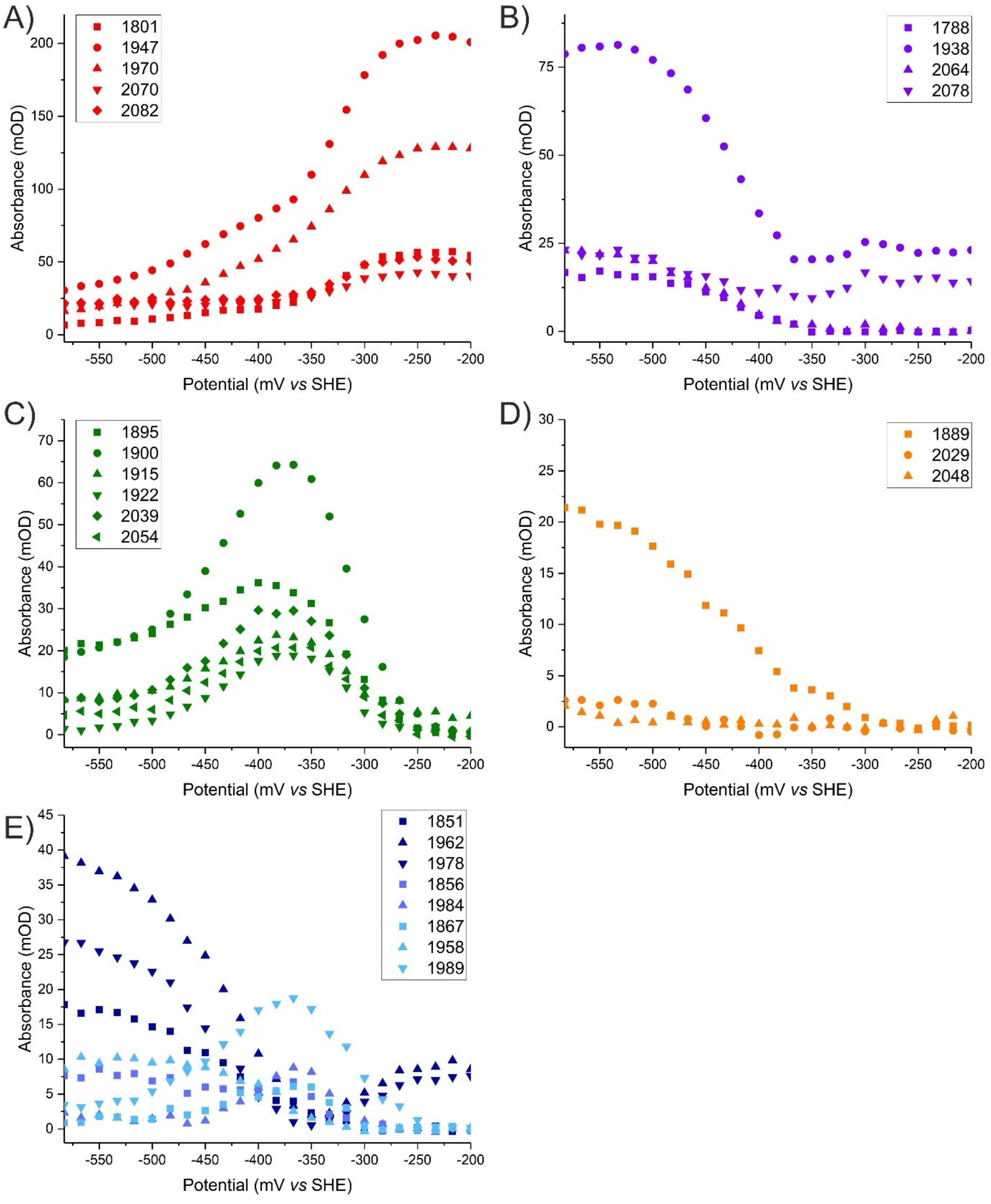

Figure S6. Titration plots for every wavenumber position as assigned to their respective species (Table 1): A) Hox; B) Hred; C) HredH^{+} family; D) HsredH^{+}; E) Hhyd family. These graphs are plotted from the same data presented in the main text, Figure 4A (a single crystal of CpI at pH 6).

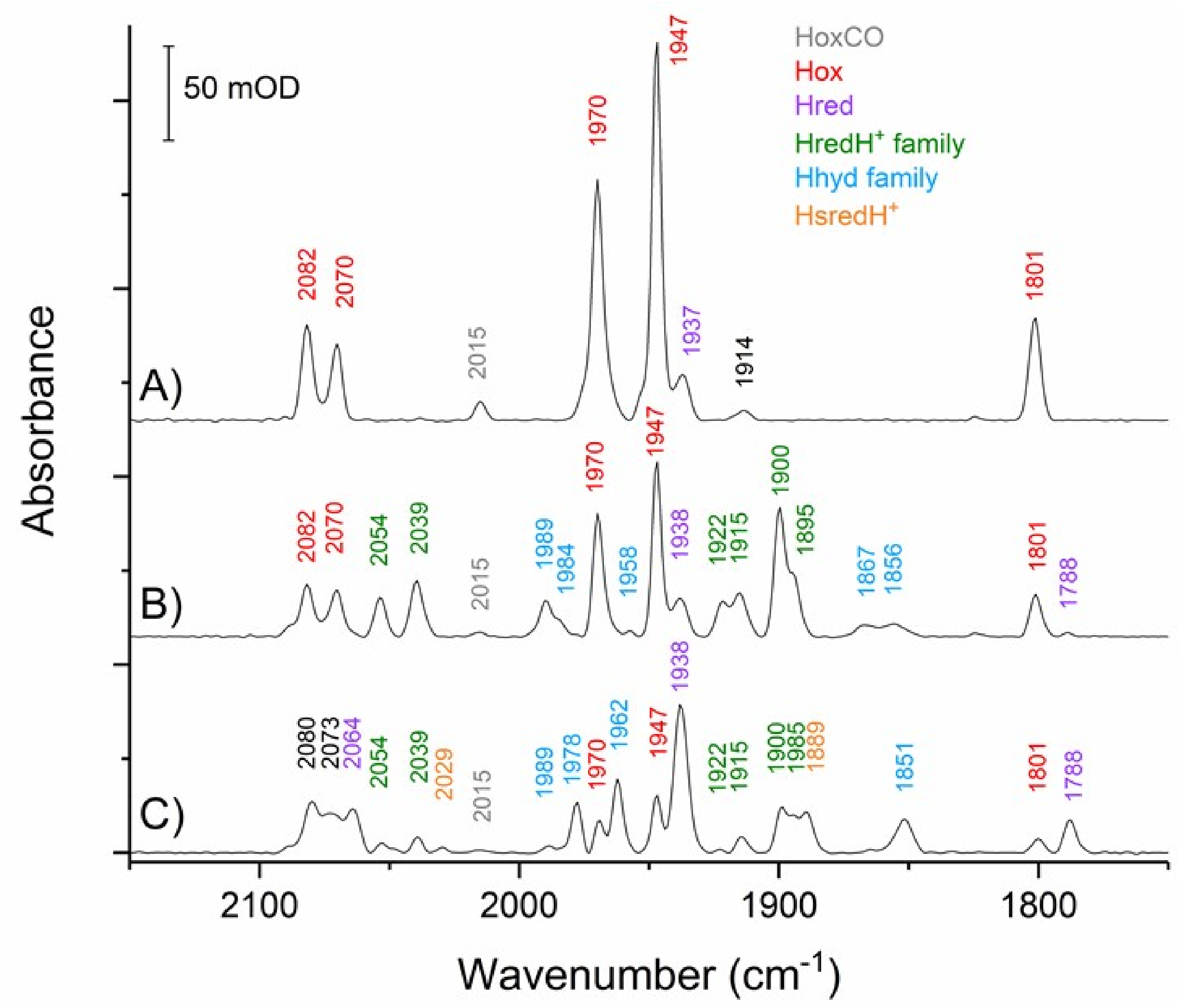

Figure S7. Spectra representative of the three regions that can be identified in the titration of CpI crystals at pH 6. A) high potential (-200 mV); B) moderate reduction (-367 mV); C) more negative potential (-583 mV). Peaks are assigned in accordance with Table 1 in the main text.

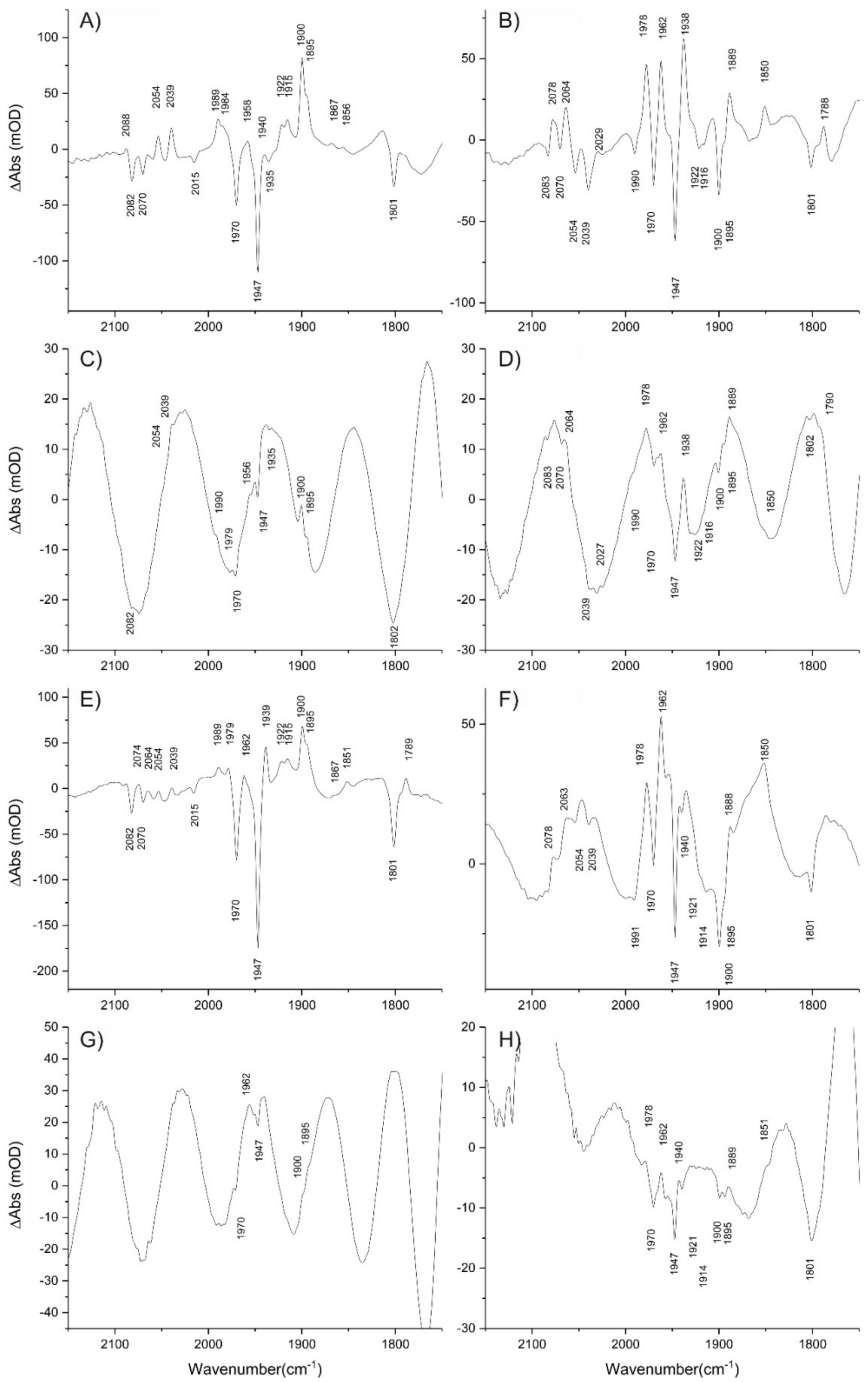

Figure S8. Difference spectra. Difference spectra from raw data were computed to complement the titration interpretation, highlighting peak depletion or accumulation without any possible bias from the baseline correction. A) CpI crystal pH 6, -367 mV *minus* -200 mV. B) CpI crystal pH 6, -583 mV *minus* -367 mV. C) CpI solution pH 6, -400 mV *minus* -200 mV. D) CpI solution pH 6, -583 mV *minus* -400 mV. E) CpI crystal pH 8, -450 mV *minus* -200 mV. F) CpI crystal pH 8, -583 mV *minus* -450 mV. G) CpI solution pH 8, -450 mV *minus* -200 mV. H) CpI solution pH 8, -583 mV *minus* -450 mV.
